# Novel influenza A(H1N2) seasonal reassortant virus identified in a patient, Sweden, April 2025

**DOI:** 10.2807/1560-7917.ES.2025.30.31.2500542

**Published:** 2025-08-07

**Authors:** Dorina Ujvari, Tove Samuelsson Hagey, AnnaSara Carnahan, Neus Latorre-Margalef

**Affiliations:** 1 Unit for Laboratory Surveillance of Viral Pathogens and Vaccine Preventable Diseases, National Influenza Center, Department of Microbiology, The Public Health Agency of Sweden, Solna, Sweden; 2 Unit for Coordination and Surveillance of Seasonal Viruses, Department of Communicable Disease Control and Health Protection, The Public Health Agency of Sweden, Solna, Sweden

**Keywords:** influenza, reassortant, H1N2

## Abstract

In April 2025, a human seasonal reassortant influenza A(H1N2) virus with a 7:1 genetic constellation was detected in Sweden in a patient seeking primary care for influenza-like illness. The neuraminidase gene of this virus was from A(H3N2) and the remaining genes from A(H1N1)pdm09. The patient recovered. No additional cases have been detected through routine surveillance. This is so far the only identified A(H1N2) reassortant among three seasonal A(H1N1)pdm09 and A(H3N2) reassortants reported in GISAID from Europe during the 2024/25 season.

A reassortant seasonal influenza A(H1N2) virus was detected in Sweden in a routine sentinel surveillance sample from an infected patient. Here we describe this case and the characterisation of the virus.

## Case description

During early April 2025 (week 14 2025) in southern Sweden, a male patient of the 40–49-year age group, without underlying conditions consulted a primary care physician following 6 days of influenza-like illness (ILI). A nasopharyngeal swab was taken within the national sentinel surveillance system [1]. The sample was forwarded to the Public Health Agency of Sweden, where the diagnosis of influenza A virus infection was established by an in-house multiplex real-time PCR targeting the matrix protein (MP) gene of influenza A and B viruses. The sample was subtyped as A(H1)pdm09 by an in-house multiplex real-time PCR targeting the haemagglutinin (HA) gene of A(H1)pdm09 and A(H3) subtypes of influenza A virus. The patient had not been vaccinated against influenza during 2023/24 or 2024/25 and had not travelled abroad in the 14 days before ILI onset. The patient did not require hospitalisation or antiviral treatment and has fully recovered.

## Influenza season 2024/25 in Sweden

**Figure 1:**
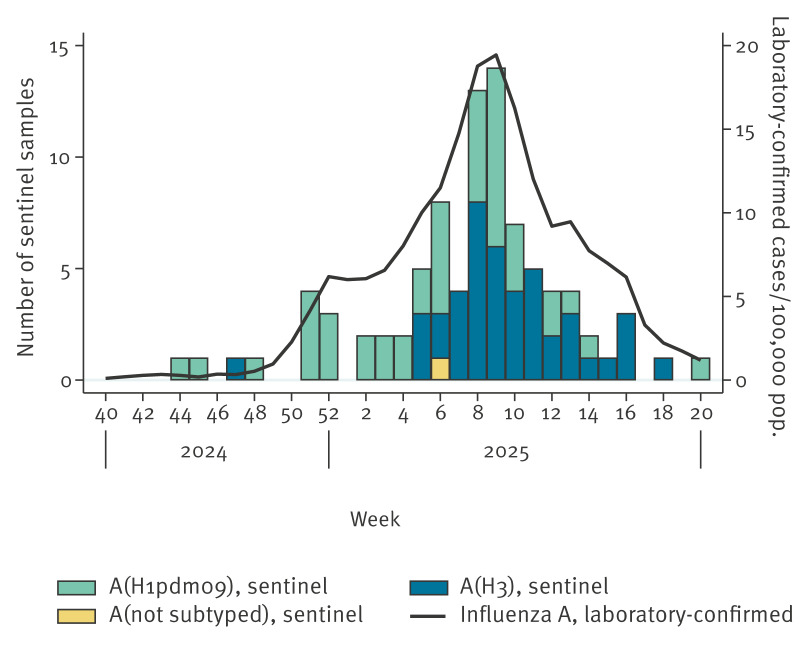
Weekly number of positive influenza A virus samples from sentinel surveillance (n = 130), by subtype, and, incidence of laboratory-confirmed influenza A cases per 100,000 population, Sweden (n = 20,494), week 40 2024–week 20 2025

According to national surveillance based on mandatory reports of laboratory-confirmed cases of influenza, the influenza epidemic in Sweden began in week 50 2024, and the number of laboratory-confirmed cases peaked in week 9 2025 (Figure 1), later than in the three previous seasons, when the peaks occurred during December. Intensity was high during weeks 8–10 of 2025 compared with historical data.

Pop.: population.

Left axis: number of influenza A positive sentinel samples, by subtype (bars); right axis: number of mandatorily reported influenza A cases per 100,000 population in Sweden (solid line). Data are presented by week of specimen collection.

In total, 24,205 laboratory-confirmed influenza cases were reported in Sweden during the surveillance period (week 40 2024–week 20 2025). Most cases were influenza A, accounting for 85% of cases in laboratory-based surveillance (20,494 of 24,205 reported cases) and 78% (130 of 167 of positive samples) in sentinel surveillance. Both A(H1N1)pdm09 and A(H3N2) influenza A subtypes circulated throughout the season. Although A(H1N1)pdm09 predominated overall, the distribution between subtypes A(H1N1)pdm09 and A(H3N2) was relatively even after week 7 2025.

Of the 152 influenza A(H1)pdm09 viruses, for which the HA gene has been sequenced so far this season, 100 belong to subclade C.1.9.3 (reference virus A/Hungary/286/2024), 20 to subclade C.1.9 (reference virus: A/Lisboa/188/2023), 30 to subclade D.3 (reference virus: A/Norway/00926/2025) and two to subclade D (reference virus: A/Victoria/4897/2022), all within clade 5a.2a, according to the *Influenza virus characterisation guidelines for the northern hemisphere influenza season 2024–2025, End of season update, issued in May 2025* (personal communication, The ECDC Respiratory Viruses and Legionella Section, European Centre for Disease Prevention and Control, June 2025). Of the 75 influenza A(H3) viruses, for which the HA gene has been sequenced, 48 belong to subclade J.2 (reference virus: A/Croatia/10136RV/2023), 20 to subclade J.2.2 (reference virus: A/Lisboa/216/2023), six to subclade J.2.1 (reference virus: A/West Virginia/51/2024) and one to subclade J.2 + N158K + K189R (reference virus: A/Netherlands/10685/2024), all within clade 2a.3a.1.

## Genetic characterisation

After being initially subtyped as A(H1)pdm09 by real-time PCR, the virus derived from the patient described in the current report was revealed on 1 May 2025 to also have A(H1N2) characteristics, following whole genome sequencing (WGS) on an Ion Torrent platform (Thermo Fisher Scientific, Waltham, Massachusetts, United States). Seven gene segments, including the HA gene of this strain derived from seasonal A(H1N1)pdm09 virus, while the neuraminidase (NA) gene segment originated from seasonal A(H3N2) virus. The genome sequence of this strain, A/Sweden/SE25–51097/2025, is available at the Global Initiative on Sharing All Influenza Data (GISAID) EpiFlu database (EPI_ISL_19857239) [2].

**Figure 2:**
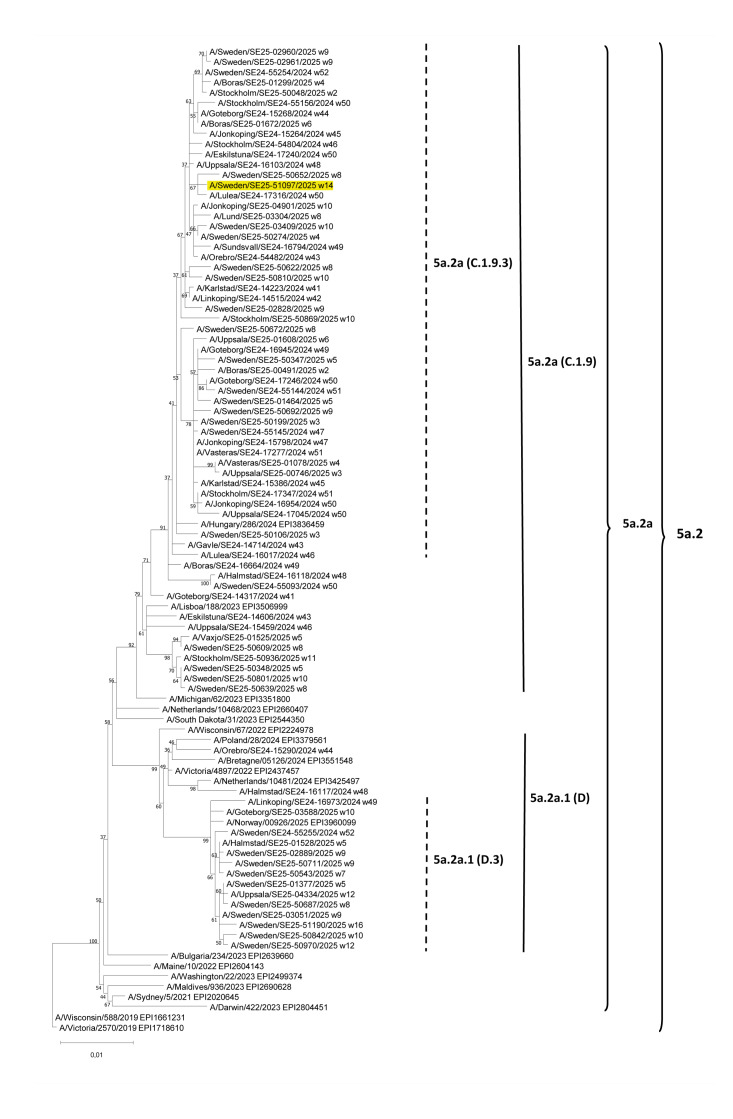
Phylogenetic analysis of the haemagglutinin genome segment of the influenza A(H1N2) virus A/Sweden/SE25–51097/2025 detected in Sweden, April 2025 (n = 96 sequences analysed)

ECDC: European Centre for Disease Prevention and Control; MEGA: Molecular Evolutionary Genetics Analysis; WHO CC: World Health Organization Collaborating Centre.

Phylogenetic analysis was performed using sequences of influenza viruses circulating in Sweden recently (i.e. week 40 2024−week 20 2025) as well as reference datasets provided by the World Health Organization Collaborating Centre (WHO CC) in London via the European Centre for Disease Prevention and Control (ECDC) for the 2024/25 season (Figure 2). The HA genome segment (EPI4310242) of A/Sweden/SE25–51097/2025 clusters with subclade C.1.9.3 (Figure 2). The HA nt sequence of the virus is not identical to any sequences of recent Swedish A(H1N1)pdm09-strains and possesses two synonym polymorphic sites, indicated by R to represent A (66%) or G (34%) and Y to represent C (25%) or T (75%) at nt positions 954 and 1539, respectively. At the HA1 and HA2 protein level, the virus has a few difference to the reference virus A/Hungary/286/2024 including amino acid substitutions V15A, V74I, V183I, A212E and V477I.

The phylogenetic tree was generated by the maximum likelihood method in MEGA11 [16]. Bootstrap values displayed on the branches are based on 500 replicates. Phylogenetic analysis is in the context of a representative selection of recent Swedish influenza A(H1N1)pdm09 viruses and a reference influenza virus dataset. Collection dates of the Swedish viruses range from 1 October 2024 to 16 April 2025. Reference datasets were provided by the WHO CC London through the ECDC for seasonal (2024/25) phylogenetic analysis. Influenza A(H1N2) virus A/Sweden/SE25–51097/2025 HA genome segment is indicated in yellow. Clades and subclades are indicated by solid line, subclades introduced in May 2025 are indicated by dashed line. The genetic distance in nt substitutions per site is indicated by the scale bar.

**Figure 3:**
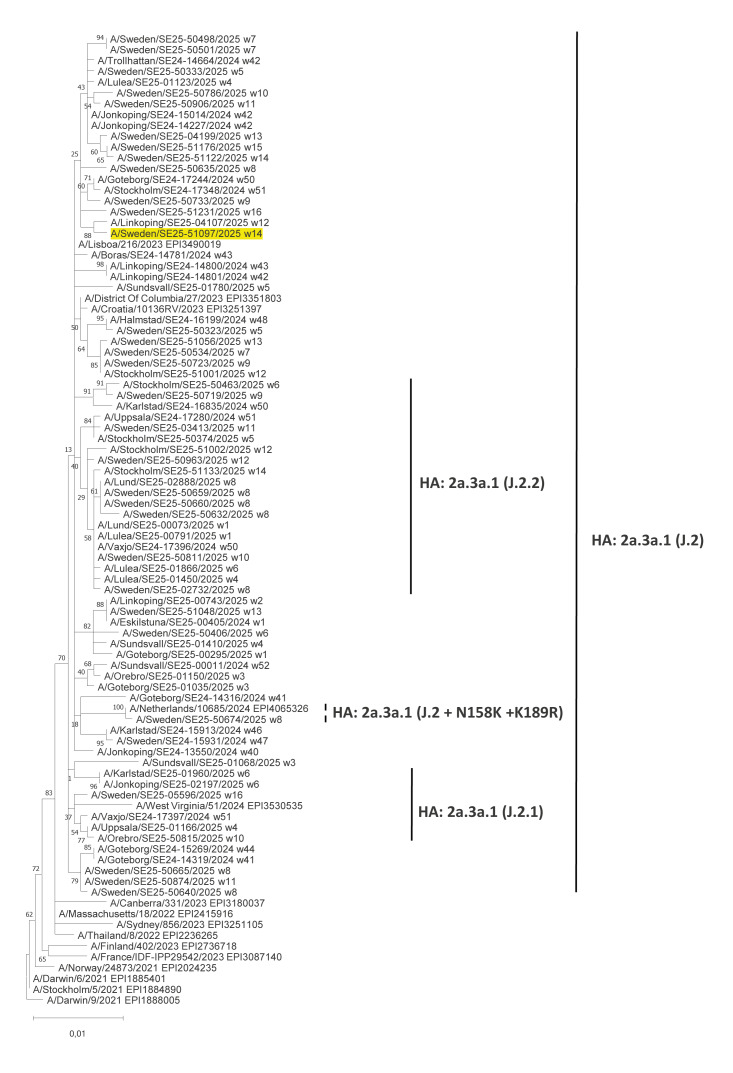
Phylogenetic analysis of the neuraminidase genome segment of the influenza A(H1N2) virus A/Sweden/SE25–51097/2025 detected in Sweden, April 2025 (n = 90 sequences analysed)

ECDC: European Centre for Disease Prevention and Control; MEGA: Molecular Evolutionary Genetics Analysis; WHO CC: World Health Organization Collaborating Centre.

In the phylogenetic analysis of the NA sequence (EPI4310240), A/Sweden/SE25–51097/2025 clusters with A(H3N2) viruses that belong to the HA subclade J.2 (Figure 3). The NA nt sequence of the virus is not identical to any of the Swedish A(H3N2) sequences identified in Sweden since October 2024. At protein level, the virus is identical with the reference virus strains A/District of Columbia/27/2023 (EPI3351803), A/Lisboa/216/2023 (EPI3490019) and A/Croatia/10136RV/2023 (EPI3251397). A/Sweden/SE25–51097/2025 does not harbour any of NA amino acid substitutions known to be associated with reduced or highly reduced sensitivity to NA inhibitors, oseltamivir, zanamivir, peramivir or laninamivir [3].

The phylogenetic tree was generated by the maximum likelihood method in MEGA11 [16]. Bootstrap values displayed on the branches are based on 500 replicates. Phylogenetic analysis is in the context of a representative selection of recent Swedish influenza A(H3N2) viruses and a reference influenza virus dataset. Collection dates of the Swedish viruses range from 1 October 2024 to 16 April 2025. Reference datasets were provided by the WHO CC London through the ECDC for seasonal (2024/25) phylogenetic analysis. Influenza A(H1N2) virus A/Sweden/SE25–51097/2025 NA genome segment is indicated in yellow. Clades and subclades are indicated by solid line, and a subclade introduced in May 2025 is indicated by dashed line. The genetic distance in nt substitutions per site is indicated by the scale bar.

The remaining six segments respectively including the MP, non-structural protein (NS), nucleoprotein (NP), polymerase basic binding protein (PB)1, PB2 and polymerase acidic protein (PA) genes were analysed by a basic local alignment search tool (BLAST) search in GISAID, and all showed highest similarities with corresponding gene segments of human A(H1N1)pdm09 viruses circulating in season 2024/25.

The M2 protein of A/Sweden/SE25–51097/2025 possesses the amino acid substitution S31N, as other characterised recently circulating influenza A viruses, which results in resistance to amantadine [4]. The PA protein of the described virus strain does not carry any of the PA amino acid substitutions known to be associated with reduced sensitivity to baloxavir [5].

In the sample, there was no evidence of mixed infection of the two seasonal influenza A virus subtypes A(H1N1)pdm09 and A(H3N2), as the sample was negative for the HA gene from A(H3N2) with real-time PCR, and all segments were the most similar to those of A(H1N1)pdm09 or A(H3N2) viruses, respectively. Furthermore, variant analysis, besides the HA gene segment, showed no ambiguous positions, which otherwise could suggest the presence of both A(H1N1)pdm09 and A(H3N2) viruses in the sample. Thus, it may be that co-infection occurred in another individual who transmitted the reassortant to the sampled patient, but no other cases have been identified within the national surveillance. These kind of transmission events are rarely captured, as previously reported in a study on severe acute respiratory syndrome coronavirus 2 (SARS-CoV-2), where sequences originating from sampled patients hospitalised in the same ward were analysed [6].

This was the only reassortant virus detected among 227 influenza A viruses characterised by WGS in Sweden during the period covering week 40 2024–week 20 2025. The case was notified to the WHO according to the International Health Regulations (IHR) and to ECDC via EpiPulse on 6 May 2025.

The risk of further transmission of the reassortant was considered low and no further public health measures were taken.

## Discussion

Only a limited number of studies have described influenza A(H1N2) reassortant viruses, harbouring an HA gene from seasonal A(H1N1) or A(H1N1)pdm09 viruses and a NA gene from seasonal A(H3N2) viruses, due to co-infections in humans. To the best of our knowledge, this is the first A(H1N2) reassortant virus detected during the 2024/25 season. During that season, two A(H3N2) reassortants were reported from the Netherlands in February 2025, with genome segments MP, NP, NS, PA and PB2 of A(H1N1)pdm09 origin and HA, NA and PB1 of A(H3N2) origin (A/Netherlands/10431/2025, EPI_ISL_19732083 and A/Netherlands/10472/2025, EPI_ISL_19732112). In earlier years, reassortant A(H1N2) influenza viruses were reported from Japan in 1981 (one case) [7], from India in 2009 (one case, A/Eastern India/N-1289/2009) [8], from the Netherlands in 2018 (one case, A/Netherlands/10407/2018) [9], from Sweden in 2019 (one case, A/Ystad/1/2018) [1], from Denmark in 2019 (one case, A/Denmark/3176/2019) [10] and A(H1N2) viruses have circulated in China in 1988/89 and worldwide in 2001 and 2003 [11-13].

The gene constellation of A/Sweden/SE25–51097/2025 is the same as in those viruses detected in Denmark and in Sweden in 2019, with seven genes (HA and the internal genes) of A(H1N1)pdm09 and an NA gene of A(H3N2) origin [1,10]. As no A(H1N2) viruses have been found in Sweden over the past 6 years, there is no reason to assume that A(H1N2) viruses have been circulating since 2019 and A/Sweden/SE25–51097/2025 is a descendant of either the Swedish or the Danish viruses identified in 2019. The A(H1N2) virus detected in India carried HA gene derived from an A(H1N1)pdm09 and the rest of the genes from an A(H3N2) virus [8]. The A(H1N2) virus from the Netherlands had a different gene constellation, with HA and NS acquired from an A(H1N1)pdm09 virus and the remaining six genes originating from an A(H3N2) virus [9]. Even though the extent of patient sampling and characterisation of influenza viruses has expanded greatly over the past 20 years, the detection of reassortant viruses has remained sporadic. This suggests either that concurrent infections with seasonal influenza A strains are not very common, or that concurrent infections might occur often, but most of reassorted A(H1N2) viruses do not usually spread easily among humans, with the exception of reassortants circulating in 1988/89 in China and worldwide between 2001 and 2003, both resulting from two distinct reassortment events, but with similar gene constellation, the HA gene of A(H1N1) and the rest of the genes of A(H3N2) origin [11-13].

Sporadic cases of zoonotic influenza viruses in humans arising from animal-to-human spillover are increasing, especially due to A(H5N1) outbreaks in domestic birds and mammals worldwide [14]. Co-infection with different seasonal and zoonotic influenza strains might lead to the formation of influenza viruses with novel constellations of gene segments. Reassortment also imposes a strong selection pressure on the virus, provoking a transient increase in the rate of amino acid replacements – a burst of positively selected adaptive changes. This might lead to emergence of variant viruses with post-reassortment adaptation to the new environment, i.e. acquiring the ability to efficiently spread among humans and cause epidemics or pandemics [15]. Therefore, it is of high importance that reference laboratories, via molecular surveillance of circulating influenza strains, can detect new reassortant viruses and that the potential public health impact of reassortant viruses is assessed.

This study has some limitations. No case finding or contact tracing was possible, so there may have been related cases that remain undetected, particularly in individuals with mild symptoms. Sampling for influenza in Sweden is confined to medically attended influenza infection, meaning those with mild or no symptoms are unlikely to be sampled. In addition, only approximately 25% of all laboratory-confirmed influenza A cases detected nationally are subtyped. As such, reassortant cases may be missed.

## Conclusion

The occurrence of the H1N2 reassortant of influenza A virus detected during routine surveillance activities in Sweden, underscores the importance for sustained systematic testing alongside genomic characterisation. Given the pandemic potential of influenza viruses of different origin, surveillance systems and analysis tools are crucial for timely and effective public health responses.

## Data Availability

All virus sequences are accessible at the Global Initiative on Sharing All Influenza Data (GISAID) EpiFlu database (EPI_ISL numbers specified in Supplementary Table 1).

## Supplementary Data

217000 Supplementary Table
