# Supplementary material for: Novel influenza A(H1N2) seasonal reassortant virus identified in a patient, Sweden, April 2025
Source: Euro Surveill. 2025 Aug 7;30(31):2500542. doi: 10.2807/1560-7917.ES.2025.30.31.2500542 (PMC12333488; doi:10.2807/1560-7917.ES.2025.30.31.2500542)
Supplement: Supplementary Table [file 2500542_Supplementary_Table1.pdf]

This supplementary material is hosted by *Eurosurveillance* as supporting information alongside the article "Novel influenza A(H1N2) seasonal reassortant virus identified in a patient, Sweden, April 2025", on behalf of the authors, who remain responsible for the accuracy and appropriateness of the content. The same standards for ethics, copyright, attributions and permissions as for the article apply. Supplements are not edited by *Eurosurveillance* and the journal is not responsible for the maintenance of any links or email addresses provided therein.

Supplementary table 1: The Swedish virus strains and their GISAID accession numbers used in the phylogenetic analysis of HA and NA gene segments

Virus strain	GISAID accession number
A/Karlstad/SE24-14223/2024	EPI_ISL_19526980
A/Goteborg/SE24-14317/2024	EPI_ISL_19527018
A/Linkoping/SE24-14515/2024	EPI_ISL_19527038
A/Eskilstuna/SE24-14606/2024	EPI_ISL_19527048
A/Gavle/SE24-14714/2024	EPI_ISL_19556249
A/Orebro/SE24-54482/2024	EPI_ISL_19556255
A/Jonkoping/SE24-15264/2024	EPI_ISL_19586470
A/Goteborg/SE24-15268/2024	EPI_ISL_19586471
A/Orebro/SE24-15290/2024	EPI_ISL_19586473
A/Uppsala/SE24-15459/2024	EPI_ISL_19586475
A/Karlstad/SE24-15386/2024	EPI_ISL_19586477
A/Jonkoping/SE24-15798/2024	EPI_ISL_19586483
A/Lulea/SE24-16017/2024	EPI_ISL_19602972
A/Uppsala/SE24-16103/2024	EPI_ISL_19602973
A/Halmstad/SE24-16117/2024	EPI_ISL_19602974
A/Halmstad/SE24-16118/2024	EPI_ISL_19602975
A/Stockholm/SE24-54804/2024	EPI_ISL_19602976
A/Boras/SE24-16664/2024	EPI_ISL_19650347
A/Sundsvall/SE24-16794/2024	EPI_ISL_19650350
A/Goteborg/SE24-16945/2024	EPI_ISL_19650352
A/Jonkoping/SE24-16954/2024	EPI_ISL_19650353
A/Linkoping/SE24-16973/2024	EPI_ISL_19650355
A/Uppsala/SE24-17045/2024	EPI_ISL_19650356
A/Eskilstuna/SE24-17240/2024	EPI_ISL_19668549
A/Goteborg/SE24-17246/2024	EPI_ISL_19668551
A/Vasteras/SE24-17277/2024	EPI_ISL_19668553
A/Lulea/SE24-17316/2024	EPI_ISL_19668555
A/Stockholm/SE24-17347/2024	EPI_ISL_19668565
A/Sweden/SE24-55093/2024	EPI_ISL_19679555
A/Sweden/SE24-55144/2024	EPI_ISL_19668557
A/Stockholm/SE24-55156/2024	EPI_ISL_19668558
A/Sweden/SE24-55145/2024	EPI_ISL_19668560

Solna Folkhälsomyndigheten, SE-171 82 Solna. Besök: Nobels väg 18. **Östersund** Folkhälsomyndigheten, Box 505, 831 26 Östersund. Besök: Campusvägen 20. Telefon 010-205 20 00 E-post info@folkhalsomyndigheten.se www.folkhalsomyndigheten.se

A/Sweden/SE24-55254/2024	EPI_ISL_19668562
A/Sweden/SE24-55255/2024	EPI_ISL_19668563
A/Boras/SE25-00491/2025	EPI_ISL_19705907
A/Stockholm/SE25-50048/2025	EPI_ISL_19705910
A/Uppsala/SE25-00746/2025	EPI_ISL_19736428
A/Vasteras/SE25-01078/2025	EPI_ISL_19769510
A/Boras/SE25-01299/2025	EPI_ISL_19769334
A/Sweden/SE25-50106/2025	EPI_ISL_19736403
A/Sweden/SE25-50199/2025	EPI_ISL_19736406
A/Vaxjo/SE25-01525/2025	EPI_ISL_19736436
A/Halmstad/SE25-01528/2025	EPI_ISL_19769339
A/Uppsala/SE25-01608/2025	EPI_ISL_19769340
A/Sweden/SE25-50274/2025	EPI_ISL_19736409
A/Sweden/SE25-01377/2025	EPI_ISL_19769343
A/Sweden/SE25-01464/2025	EPI_ISL_19769344
A/Sweden/SE25-50347/2025	EPI_ISL_19769429
A/Sweden/SE25-50348/2025	EPI_ISL_19769430
A/Boras/SE25-01672/2025	EPI_ISL_19769431
A/Sweden/SE25-50543/2025	EPI_ISL_19782063
A/Sweden/SE25-50609/2025	EPI_ISL_19782068
A/Sweden/SE25-50622/2025	EPI_ISL_19782069
A/Sweden/SE25-50639/2025	EPI_ISL_19782075
A/Sweden/SE25-50652/2025	EPI_ISL_19782078
A/Sweden/SE25-02828/2025	EPI_ISL_19787364
A/Sweden/SE25-02889/2025	EPI_ISL_19787365
A/Sweden/SE25-02960/2025	EPI_ISL_19787366
A/Sweden/SE25-02961/2025	EPI_ISL_19787367
A/Sweden/SE25-03051/2025	EPI_ISL_19787369
A/Sweden/SE25-50672/2025	EPI_ISL_19787373
A/Sweden/SE25-50687/2025	EPI_ISL_19787375
A/Sweden/SE25-50692/2025	EPI_ISL_19787376
A/Sweden/SE25-50711/2025	EPI_ISL_19787377
A/Lund/SE25-03304/2025	EPI_ISL_19814430
A/Goteborg/SE25-03588/2025	EPI_ISL_19814397
A/Stockholm/SE25-50869/2025	EPI_ISL_19814399
A/Sweden/SE25-03409/2025	EPI_ISL_19814402
A/Sweden/SE25-50801/2025	EPI_ISL_19814406
A/Sweden/SE25-50810/2025	EPI_ISL_19814408
A/Sweden/SE25-50842/2025	EPI_ISL_19814409

A/Stockholm/SE25-50936/2025	EPI_ISL_19857209
A/Uppsala/SE25-04334/2025	EPI_ISL_19857212
A/Sweden/SE25-50970/2025	EPI_ISL_19857217
A/Sweden/SE25-51097/2025	EPI_ISL_19857239
A/Jonkoping/SE25-04901/2025	EPI_ISL_19857228
A/Sweden/SE25-51190/2025	EPI_ISL_19857236
A/Jonkoping/SE24-13550/2024	EPI_ISL_19526971
A/Jonkoping/SE24-14227/2024	EPI_ISL_19526997
A/Goteborg/SE24-14316/2024	EPI_ISL_19527008
A/Goteborg/SE24-14319/2024	EPI_ISL_19527028
A/Trollhattan/SE24-14664/2024	EPI_ISL_19556248
A/Boras/SE24-14781/2024	EPI_ISL_19556250
A/Linkoping/SE24-14800/2024	EPI_ISL_19556251
A/Linkoping/SE24-14801/2024	EPI_ISL_19556252
A/Jonkoping/SE24-15014/2024	EPI_ISL_19586466
A/Goteborg/SE24-15269/2024	EPI_ISL_19586472
A/Karlstad/SE24-15913/2024	EPI_ISL_19602969
A/Halmstad/SE24-16199/2024	EPI_ISL_19602977
A/Sweden/SE24-15931/2024	EPI_ISL_19602978
A/Karlstad/SE24-16835/2024	EPI_ISL_19668546
A/Goteborg/SE24-17244/2024	EPI_ISL_19668550
A/Uppsala/SE24-17280/2024	EPI_ISL_19668554
A/Stockholm/SE24-17348/2024	EPI_ISL_19668568
A/Vaxjo/SE24-17396/2024	EPI_ISL_19679550
A/Vaxjo/SE24-17397/2024	EPI_ISL_19679551
A/Sundsvall/SE25-00011/2024	EPI_ISL_19679554
A/Lund/SE25-00073/2025	EPI_ISL_19680741
A/Goteborg/SE25-00295/2025	EPI_ISL_19705900
A/Eskilstuna/SE25-00405/2024	EPI_ISL_19706039
A/Linkoping/SE25-00743/2025	EPI_ISL_19705920
A/Lulea/SE25-00791/2025	EPI_ISL_19705921
A/Goteborg/SE25-01035/2025	EPI_ISL_19736430
A/Sundsvall/SE25-01068/2025	EPI_ISL_19736431
A/Lulea/SE25-01123/2025	EPI_ISL_19769332
A/Uppsala/SE25-01166/2025	EPI_ISL_19769333
A/Orebro/SE25-01150/2025	EPI_ISL_19736434
A/Sundsvall/SE25-01410/2025	EPI_ISL_19769336
A/Lulea/SE25-01450/2025	EPI_ISL_19769338
A/Sweden/SE25-50323/2025	EPI_ISL_19769347

A/Sweden/SE25-50333/2025	EPI_ISL_19769348
A/Sundsvall/SE25-01780/2025	EPI_ISL_19769432
A/Stockholm/SE25-50374/2025	EPI_ISL_19769434
A/Lulea/SE25-01866/2025	EPI_ISL_19769435
A/Karlstad/SE25-01960/2025	EPI_ISL_19769436
A/Jonkoping/SE25-02197/2025	EPI_ISL_19769449
A/Sweden/SE25-50406/2025	EPI_ISL_19769450
A/Stockholm/SE25-50463/2025	EPI_ISL_19769451
A/Sweden/SE25-50498/2025	EPI_ISL_19782059
A/Sweden/SE25-50501/2025	EPI_ISL_19782060
A/Lund/SE25-02888/2025	EPI_ISL_19782064
A/Sweden/SE25-50534/2025	EPI_ISL_19782065
A/Sweden/SE25-50632/2025	EPI_ISL_19782071
A/Sweden/SE25-50635/2025	EPI_ISL_19782073
A/Sweden/SE25-50640/2025	EPI_ISL_19782076
A/Sweden/SE25-02732/2025	EPI_ISL_19787363
A/Sweden/SE25-50659/2025	EPI_ISL_19787370
A/Sweden/SE25-50660/2025	EPI_ISL_19787371
A/Sweden/SE25-50665/2025	EPI_ISL_19787372
A/Sweden/SE25-50674/2025	EPI_ISL_19787374
A/Sweden/SE25-50719/2025	EPI_ISL_19787378
A/Sweden/SE25-50723/2025	EPI_ISL_19787379
A/Sweden/SE25-50733/2025	EPI_ISL_19787380
A/Orebro/SE25-50815/2025	EPI_ISL_19814369
A/Sweden/SE25-03413/2025	EPI_ISL_19814403
A/Sweden/SE25-50786/2025	EPI_ISL_19814404
A/Sweden/SE25-50811/2025	EPI_ISL_19857207
A/Sweden/SE25-50874/2025	EPI_ISL_19814410
A/Sweden/SE25-50906/2025	EPI_ISL_19814411
A/Linkoping/SE25-04107/2025	EPI_ISL_19857208
A/Sweden/SE25-04199/2025	EPI_ISL_19857213
A/Sweden/SE25-50963/2025	EPI_ISL_19857216
A/Stockholm/SE25-51001/2025	EPI_ISL_19857221
A/Stockholm/SE25-51002/2025	EPI_ISL_19857222
A/Sweden/SE25-51056/2025	EPI_ISL_19857238
A/Sweden/SE25-51097/2025	EPI_ISL_19857239
A/Stockholm/SE25-51133/2025	EPI_ISL_19857230
A/Sweden/SE25-51048/2025	EPI_ISL_19857281
A/Sweden/SE25-51122/2025	EPI_ISL_19857232

A/Sweden/SE25-51176/2025	EPI_ISL_19857234
A/Sweden/SE25-05596/2025	EPI_ISL_19884736
A/Sweden/SE25-51231/2025	EPI_ISL_19884739
